# A New Interprofessional Community-Service Learning Program, HATS (Health Ambassador Teams for Seniors) to Improve Older Adults Attitudes about Telehealth and Functionality

**DOI:** 10.3390/ijerph181910082

**Published:** 2021-09-25

**Authors:** Donald Jurivich, Carter Schimke, Dakota Snustad, Mitchell Floura, Casey Morton, Marsha Waind, Jeremy Holloway, Sclinda Janssen, Meridee Danks, Karen Semmens, Gunjan Dhawan Manocha

**Affiliations:** 1 Department of Geriatrics, School of Medicine and Health Sciences, University of North Dakota, Grand Forks, ND 58202, USA; carter.schimke@und.edu (C.S.); dakota.snustad@und.edu (D.S.); mitchell.floura@und.edu (M.F.); casey.morton@und.edu (C.M.); marsha.waind@ndus.edu (M.W.); jeremy.holloway@und.edu (J.H.); gunjan.manocha@und.edu (G.D.M.); 2 Department of Occupational Therapy, School of Medicine and Health Sciences, University of North Dakota, Grand Forks, ND 58202, USA; sclinda.janssen@und.edu; 3 Department of Physical Therapy, School of Medicine and Health Sciences, University of North Dakota, Grand Forks, ND 58202, USA; meridee.danks@und.edu; 4 Department of Nursing, College of Nursing and Professional Disciplines, University of North Dakota, Grand Forks, ND 58202, USA; karen.semmens@und.edu

**Keywords:** community service learning, older adult population health, telehealth, geriatric assessment

## Abstract

Senior population health often is underrepresented in curricula for medical and allied health students. Furthermore, entrenched and dense curricular schedules preclude interprofessional teams from clinical experiences related to senior population health. Community service learning potentially offers the opportunity to engage interprofessional students with a panel of older adults to assess health promotion metrics over time. To test this educational concept, we created Health Ambassador Teams for Seniors, also known as HATS. Utilizing a telehealth platform, interprofessional student teams were tasked with older adult wellness promotion. The annual Medicare wellness exam served as a template for patient encounters which was enhanced with key elements of geriatric assessment such as gait and balance, cognition, and functional evaluations. The objective was to have dyads of interprofessional students conduct telehealth visits and gather healthcare data to be used for serial patient encounters and track functional trajectories over time. As a proof of concept, pilot telehealth encounters with medical, physical therapy, nursing and occupational therapy students revealed that data on older adult functional performances such as gait speed, Timed Up and Go test (TUG), and Mini-Cog test could be acquired through telehealth. Equally importantly, trainees received diverse feedback from faculty, peers and volunteer patients. A Research Electronic Data Capture (REDCap) data repository allows trainees to track patient trends relative to their health promotion recommendations as well as handoff their patient panel to the next set of trainees. The HATS program promises to strengthen the Geriatric Workforce, especially with senior population health.

## 1. Introduction

### 1.1. Community-Based Service Learning

With changing health care complexity in the United States and globally, more allied health and medical schools look to high impact experiences such as community-service or service-learning to address student-centered curriculum [1,2,3,4,5,6,7,8]. Community-based service learning (CSL) provides a curricular avenue to strengthen student learning, civic responsibility, professionalism and sense of community [9,10]. CSL programs often circumvent the limitations of time and space associated with direct clinical placements of medical and allied health profession trainees. Such programs give the trainees opportunities to interact with a range of educators and patients, potentially enhancing education around social determinants of health [10,11,12,13].

The CSL programs also provide a reciprocal partnership between the academic institutions and community members [14]. Although limited data is available on how service-learning impacts communities, current programs often address medical needs of under-resourced populations [2,15,16]. Many programs report highly positive experiences among community members interacting with students and educators in medical education programs [7,17,18,19,20].

While community service-learning can help fill gaps in underserved populations, it can be a vehicle to train and better prepare health care professional students’ in Geriatrics. With the rapid and steady rise of older adult populations [21], non-specialized care providers will have an increasing percentage of patients who are 65 years old and older. Some medical schools have community–based older adult encounters, but none of these are designed to promote the health of the older adult volunteer [5,22]. Another gap and opportunity of community service programs is to inculcate learning about interprofessional teams [4].

### 1.2. Project Rationale

The number of older adults is steadily growing and necessitates a healthcare workforce that can competently care for a population that acquires more chronic conditions and functional limitations over time [23,24]. With this population growth, the demand for Geriatricians is expected to increase 45% by the year 2025, yet the emerging number of Geriatricians throughout the country does not match this expected growth [25]. The number of Geriatricians in America actually decreased from 10,270 in 2000 to 8502 in 2010 [26]. Given the shortage of global geriatric expertise, current educational efforts seek to better train the primary care workforce in older adult health care [27]. Geriatric principles of care can prevent functional decline, better align health care with what matters to older adults, and substantially reduce health care costs [24]. Thus, strengthening geriatrics knowledge among the next generation of health care workers is an important goal [27].

The key competencies for designing a geriatric educational intervention include knowledge and appreciation of older adult’s functional status in addition to medical issues [28]. Health care workers commonly think in terms of chronic conditions rather than geriatric syndromes such as falls, failure to thrive, and frailty [29,30,31]. Additionally, they frequently focus on curing disease rather than points in time appropriate for palliative care [24,31]. To enhance medical trainee’s knowledge and attitudes about older adult care, a program like Health Ambassadors Teams for Seniors (HATS) can introduce students to the full spectrum of elderly health status ranging from healthy to terminally ill older adults. Equally important, the HATS program can hone student communication skills with other health care providers. This inter-relationship is essential to multidisciplinary teams so they may properly execute Comprehensive Geriatric Assessments (CGA). Finally, the HATS program provides a new service to older adults, driven by the geriatrics expertise of faculty mentors. Through scripted protocols, student teams generate geriatric assessments that can be communicated to primary care providers so they can address previously unfounded problems. Indeed, geriatric assessments often unearths 2–3 new problems that are not detected in usual health care [32].

## 2. Methods

### 2.1. HATS Protocol

The Health Ambassador Teams for Seniors (HATS) program adapts telehealth encounters for older adults with interprofessional student teams tasked with assessing cognitive, psychological, and physical function of their patients. A workgroup of interprofessional faculty and students teamed together to review and adapt the Annual Wellness Exam (AWE) [33] protocol for telehealth assessments and incorporated Age-Friendly Healthcare framework of the Geriatric 4Ms (What Matters, Mentation, Mobility, and Medications) into the protocol [28]. The modified Medicare Annual Wellness Exam was developed for an interprofessional team of students as a scripted assessment to be performed on volunteer older adults through Zoom meetings. The list of assessments aligned with the Geriatric 4M’s are presented in Table 1.

### 2.2. Structured Patient Visits and Instruction

The first priority of creating the HATS program was to generate a cadre of older adult volunteers. To do so, we enlisted community–based professionals associated with the medical and allied health departments of the university. These professionals conduct home visits and are able to recruit patient volunteers. This arrangement provides a patient panel for the training program with a range of functional impairments either cognitively, physically, or both. The advantage of recruitment by a community–based healthcare professional is that a health professional can facilitate a telehealth visit with the older adult while they are conducting a home visit. The logistics of aligning student teams with a visiting home health professional and their patients requires some administrative effort towards the scheduling process. To further expand the cadre of volunteer patients the HATS program used advertisements and word of mouth referrals to enhance the range of volunteers from functionally impaired to functionally intact older adults.

Inclusion criteria for older adult volunteers includes any community dwelling 65 years or older adult with 2 or more chronic conditions, able and willing to communicate via telehealth, English-speaking, and non-terminal. Exclusion criteria for the program includes those who have no underslying conditions (are healthy) or are terminally ill, non-English speaking, have unresolved pain, currently reside in nursing home/hospice or, are unwilling to participate in telehealth. Because of ease and familiarity, the Zoom format was chosen for performing all telehealth visits, with telephone contact reserved as a backup. Home health agencies involved in this work would identify potential clients, obtain consent from the older adults, and connect them to the HATS program logistics team. Based on older adult availability and student/faculty schedule, the telehealth visit would be scheduled. A home health aide from the community-based health care agency is present during telehealth visits to assist the older adults with technical issues.

Training materials were developed to introduce interprofessional student teams to the AWE and how to professionally conduct the exam through telehealth. Student teams were formed in pairs of students, each from a different health discipline. They were instructed to conduct an AWE on each other to familiarize themselves with the protocol. Once prepared, the student teams engaged with an older adult volunteer and split the interview with one student starting the AWE and then handing off the AWE to the other student midway in the process. The students summarized their assessments for the older adult and inquired as to whether the older adult volunteer wanted a report sent to their primary care doctor. Students were asked to formulate an assessment and plan for each patient using the Geriatric 4Ms of What Matters, Medications, Mentation, and Mobility [28]. They wrote the assessment and plan from their AWE and presented it to a faculty member for review and suggestions. The students were asked to back up their plan with evidence-based medicine and citations from the medical literature, especially from large randomized controlled trials.

In addition to constructing a geriatric assessment and plan for each patient, trainees were asked to input data about health values from each patient such as vital signs, gait speed or TUG, cognitive screens and other elements from the AWE. These data were stored in a secure web-based repository called REDCap for future population health analyses and program impact.

Trainees were asked to create a communication plan with their volunteer patients and identify health promotion plans, methods of engagement, and adherence. In this sense, the student teams act as health coaches or ambassadors for health promotion.

Debriefings: Faculty-student team discussions followed the AWE and periodic follow ups with the patients. During these sessions, students had a 360-degree evaluation that included self-evaluation, peer assessment, and faculty input. Over time, as patient panels accrue, additional meetings were scheduled to evaluate how well student teams are influencing the health metrics of their patients. These metrics included changes such as the average number of steps patients take weekly, the percent of patients engaged in twice weekly resistance training, average systolic blood pressure, gait speed and other metrics such as fall rates. As the program matures, student teams will be able to compare each other’s patient panel metrics and determine which team is most effective in health promotion.

During the debriefings, student teams discussed the advantages and the disadvantages of using telehealth, differentiate between telehealth and telemedicine, and discuss the telepresenters’ role. Throughout the program, the students analyzed the strengths and limitations of patient care via telehealth. Following the telehealth visit, students and older adults are asked to complete a feedback survey on their experience with telehealth via Qualtrics or paper-based surveys.

## 3. Results

### 3.1. Proof of Concept

This educational pilot produced both unexpected and expected results. The project’s proof of concept was fulfilled in that student teams were successfully paired with functionally impaired older adult volunteers living in the community. Unexpected findings include the facilitation of telehealth encounters with community–based direct care workers who perform home visits. This result contrasts with usual clinical training practices of utilizing university rather than community-based personnel for medical education. Although we did not conduct a formal qualitative analysis of direct care worker attitudes about HATS, most of the feedback was highly positive and the health care workers thought that their clients (patients) benefited from the experience as did they. An additional unexpected finding was the turnover of direct health care workers in the community setting, potentially associated with the pandemic. We note nearly 25% primary care provider turnover in some of our clinical training sites since the local COVID-19 surge of early 2021, thus it is not surprising that allied health professions experience similar turnover, either due to retirements or change of health system affiliation.

### 3.2. Feedback Surveys for Older Adults Were Well Received

**Figure 1:**
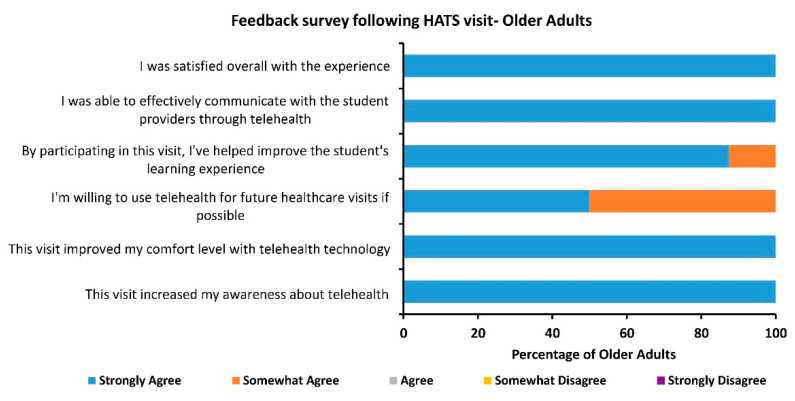
Results of the feedback survey given to participating older adults following the HATS telehealth visit.

HATS program was launched in spring 2021 and has completed 9 HATS visits during the initial proof-of-concept phase of the program. Based on this success the program anticipates 4 HATS visits scheduled weekly during its implementation phase. A paper-based survey was provided to each older adult following the visit that asked qualitative questions on their attitude towards telehealth and specific interactions with students throughout this experience. Out of 9 visits, 8 older adults completed the surveys. A summary of the survey results is presented in Figure 1. There were 6 questions based on a 5-point Likert scale ranging from “strongly agree” to “strongly disagree”. Additionally, 2 descriptive questions were asked, the first being “Please tell us what went well during this visit” and the second being “Please tell us where we can improve for future visits”. All 8 older adults liked/enjoyed their telehealth encounters and did not suggest any areas for improvement.

### 3.3. Strengths and Weakness of the HATS Program

The HATS program was launched as a learning activity for students but as more visits have been set up, the program unexpectedly transformed into a crucial community-service learning opportunity. Additional home health agencies have become involved and the program has reached several rural areas of North Dakota in addition to the urban based program. The biggest strength of this work is the inclusion of inter-professional teamwork as uniquely applied to a telehealth setting. The other strength includes inclusion of Age-Friendly and Geriatric 4Ms education at an early stage for various healthcare trainees. However, as with any project, this work also has its weaknesses. These include exclusion of patients living alone or at skilled nursing facilities, inaccessibility to some populations in rural setting due to poor internet access, and the lack of computers or long-distance travel for home health aides. Additionally, the project entails prospective observation and while we are able to track functional trajectories over time with enrolled older adults, we do not have a control “non-telehealth” group to see if this innovative telehealth intervention changed the functional trajectory of older adults over time as compared to an in-person intervention.

Because the HATS program engaged only English-speaking participants, this report has a bias that needs to be addressed in future studies that include older adults who speak English as their second language or non-English speakers. Our past experience with getting interpreters for telehealth is a consistent barrier in terms of cost and availability. Additionally, noted is the under-representation of Native Americans in this project even though 8% of the project’s catchment is populated by different tribal nationalities, several of which may have cultural preferences and concerns about telehealth, especially within multi-generational households.

Although we find that the HATS program is both feasible and well received by both older adults and students, we recognize the biases and limitations of a pilot sample size for both quantitative and qualitative data. One of the clear biases was older adult recruitment which relied upon proprietary home health agencies and family members of faculty. Thus, building a registry of older adult volunteers will need more expansive efforts particularly directed towards under resourced populations, including refugees. The lack of PTSD assessments in our protocol may miss key elements of the AWE regarding veteran and new citizen participants, both which have higher PTSD levels than the population at large [41]. 

## 4. Discussion

### 4.1. HATS Assessment Protocol

The key result of this interprofessional collaborative is the successful creation of a community service- based program called Health Ambassador Teams for Seniors (HATS). The new program fills an education gap on how to assess and manage the older adult patient population. The program was originally conceptualized with student teams conducting visits at older adult homes, however, the pandemic shifted patient encounters to a telehealth platform. One objective of this program is to enhance history taking skills and obtain feedback from peers, faculty, and volunteer patients. For pre-clinical trainees this format provides a real rather than simulated patient care experience before they embark on clinical rotations. The patient encounter is structured around an annual wellness exam that includes key geriatric assessments such as memory and balance evaluations. Both of these physical exam elements are rarely practiced in usual clinical encounters with patients, thus older adults are introduced to new methods of evaluating their health status such as risk for falls and memory loss.

The educational experience has two major components: (i) clinical encounters with older adult volunteers and (ii) data collection and assessment of health trends among the population of older adult participants. Various Public Health Competencies (PHC) are linked to the students’ collection of health metrics through their patient encounters. PHC1 is understanding how to use biostatistics for better health outcomes [42]. As an example related to this project, students track the percentage of their patients who meet systolic blood pressure values advocated by the Systolic Blood Pressure Intervention Trial (SPRINT) study [43]. PHC2 entails epidemiology whereby students assess and evaluate population trends in chronic conditions and geriatric syndromes [42]. For example, for each patient panel assigned, students need to track the number of falls and falls with injury amongst their older adult population. PHC3 embraces environmental influences on health. Both the home and telehealth visits provide an opportunity for students to evaluate the home environment, especially for safety and fall risk assessment. PHC4 entails health policy and management [42]. This competency is cultivated in the program by student teams examining local programs for older adults such as Stepping On, Bone Builders and other evidence-based health promotion community programs [44,45]. They also explore internet-based resources for older adult health promotion via the State of North Dakota website, ndc3.org (last accessed on 23 September 2021), which aligns older adults with health promotion programs in their area. PHC5 examines how social, behavioral, and cultural matters contribute to public health issues among older adults [42]. During the patient encounters, students learn about social support systems for older adults and how each patient’s social network is structured, or not. 

**Figure 2:**
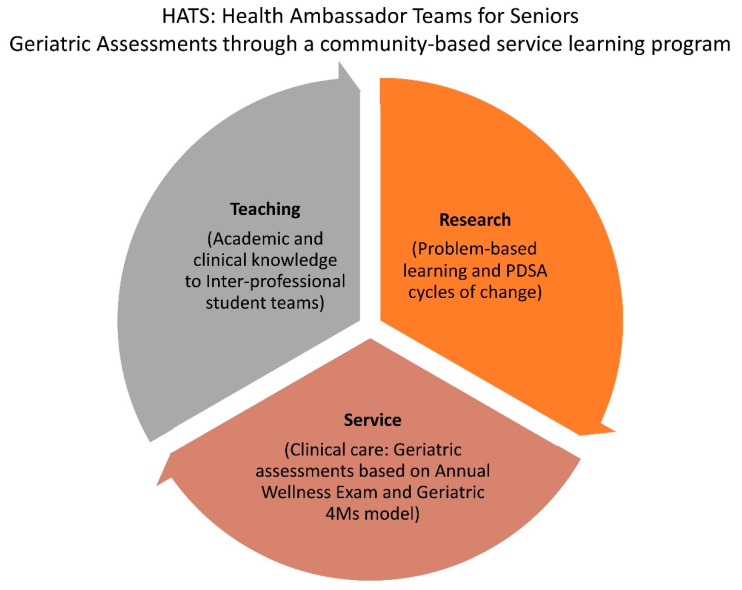
Relationship of HATS program to teaching, scholarly works and community service [46].

Some of the ways that the HATS program intends to deliver population health competencies include student collaborations to envision improved health of patients and evidence—based methods for empowering them to make healthy decisions (Figure 2). Furthermore, the program fosters interprofessional teams and competencies linked to interprofessional education.

### 4.2. IPE Competencies

The four main competencies of interprofessional education introduced by the Interprofessional Education Collaborative’s (IPEC) most recent report [47] are:

1. Work with individuals of other professions to maintain a climate of mutual respect and shared values.
2. Use the knowledge of one’s own role and those of other professions to appropriately assess and address the health care needs of patients and to promote and advance the health of populations.
3. Communicate with patients, families, communities, and professionals in health and other fields in a responsive and responsible manner that supports a team approach to the promotion and maintenance of health and the prevention and treatment of disease.
4. Apply relationship-building values and the principles of team dynamics to perform effectively in different team roles to plan, deliver and evaluate patient/population-centered care and population health programs and policies that are safe, timely, efficient, effective, and equitable.

The HATS program aims to effectively improve student abilities in each of the four domains. By performing the Geriatric Annual Wellness Exam as an interprofessional cohort, students work with each other in a manner that builds mutual respect (Competency 1). By working together towards the common goal of providing a Personalized Prevention Plan (PPP) each student uses the knowledge of their own role to assess the needs of the geriatric participant and promote geriatric population health (Competency 2). Each student participates in the generation of the older adult’s PPP, thus improving geriatric health outcomes (Competency 3). Lastly, student teams ascertain the efficiency, effectiveness, and equity of interprofessional patient/population-centered care, especially as they assess the impact of their efforts on stabilizing or improving older adult’s functional status (Competency 4).

### 4.3. Health Disparities

One goal of the HATS program is to teach students about health disparities and provide avenues to health equity solutions, particularly as they relate to aging. Recently, the National Institute on Aging (NIA) developed a framework that emphasizes the importance of further research into health disparities [48]. This framework organizes health disparities and population-health differences into sociocultural, economic, behavioral, environmental, and biological factors. Some specific examples of pertinent health disparities among older adults include geographic disparities and racial disparities. The extension of the program to rural and Native American regions will help diversify patient panels. Eventually, data from the REDCap registry can be analyzed according to subpopulations to determine if student teams are impacting the health metrics of under–resourced older adults.

Lastly, ageism is a prevalent issue that can result in decreased access to care and negative health outcomes [49,50]. The HATS program will introduce health professional students to the geriatric population earlier in their education. This early introduction may help stymie ageism in tomorrow’s health care providers.

## 5. Conclusions

Given that only a fraction of older receive an annual wellness exam or a geriatric assessment [51], the HATS program offers a new community service for the older adult population that can potentially benefit health promotion amongst older adults while simultaneously honing the population health skills of medical trainees. Student team encounters with older adult volunteers employ a template for an annual wellness exam modified to include key elements of geriatric assessment such as gait and balance, cognition, and functional evaluations. The program objectives include direct clinical experiences for interprofessional teams, thus introducing students to Geriatrics, Health Promotion, Population Health, Telemedicine and Interprofessional health care. The scope of competencies is admittedly broad, but decidedly fills gaps in current educational offerings. Currently, few if any medical training programs have trainees gather healthcare data to be used for serial encounters with their patients so as to track their functional trajectories. Thus, for the first time, trainees will have an opportunity to see how their health care plans “move the needle” towards better functional outcomes in the aging population. An aspirational goal of the HATS program is to improve student engagement with older adults and through increased experiential knowledge become interested in Geriatrics and older adult health care as specialists and future educators.

## Figures and Tables

**Table 1:** Assessments during HATS visit as aligned with Geriatric 4Ms.

Geriatric 4M’s	Tests Utilized in Assessment
What Matters	(1) Questions on patient priorities for health and daily activities of living (e.g., “What would you like us to know about you?”, “What matters most to you about your health?”) [28] (2) Functional ability and quality of life through the Katz Activities of Daily Living (ADLs) and Lawton-Brody Instrumental Activities of Daily Living (IADLs) [34]
Medications	Medication reconciliation accompanied by cross-screening with Beer’s Criteria for potentially inappropriate medications [35]
Mentation	Patient Health Questionnaire (PHQ-2) questionnaire for depression [28,36], Mini-Cog for cognitive impairment [37]
Mobility	(1) Timed-Up and Go (TUG) test [38], (2) Five-times sit-to-stand (5xSTS) [39], or (3) Four-stage balance test (FSBT) to measure fall risk [40]
